# Relationship between Interleukin-10 −1082A/G Polymorphism and Risk of Ischemic Stroke: A Meta-Analysis

**DOI:** 10.1371/journal.pone.0094631

**Published:** 2014-04-14

**Authors:** Jun Jin, Wuying Li, Lingmei Peng, Jian Chen, Rong Li, Peihua Wu, Sheng Tan

**Affiliations:** 1 Department of Neurology, Zhujiang Hospital of Southern Medical University, Guangzhou, China; 2 Department of Neurology, The First People's Hospital of Foshan, Foshan, China; 3 Department of Neurology, Zhumadian zhongxin Hospital, Zhumadian, China; INRCA, Italy

## Abstract

**Objective:**

To analyze the association between −1082A/G polymorphism in interleukin-10 (IL-10) gene and ischemic stroke (IS) risk by meta-analysis.

**Methods:**

We carried out a systematic electronic search in PubMed, BIOSIS Previews, Science Direct, Chinese National Knowledge Infrastructure, Chinese Biomedical Database, Weipu database and WANGFANG Database. Pooled odds ratios (ORs) with 95% confidence intervals (95%CIs) were calculated to assess the strength of the association.

**Results:**

7 studies were included. There was no significant association between IL-10 −1082A/G polymorphism and IS risk under all genetic models in overall estimates (A vs. G: OR = 1.23,95%CI = 0.85–1.79;AA vs. GG: OR = 1.01,95%CI = 0.47–2.19; AG vs. GG: OR = 0.76, 95%CI = 0.38–1.55; AA+AG vs. GG: OR = 0.89,95%CI = 0.46–1.73; AA vs. AG+GG: OR = 1.39, 95%CI = 0.91–2.13). Similarly, no associations were found in subgroup analysis based on ethnicity and source of controls. However, removing the study deviating from Hardy–Weinberg equilibrium (HWE) produced statistically significant associations for overall estimates under recessive model(AA VS. AG+GG OR 1.58, 95% CI 1.04–2.42) and among Asians in all genetic models (A VS.G OR 1.64, 95% CI 1.07–2.53; AA vs. GG OR1.91, 95% CI 1.31–2.80; AG vs. GG OR1.44, 95% CI 1.09–1.91; AA+AG vs. GG OR 1.54, 95% CI 1.18–2.01;AA VS. AG+GG OR 1.79, 95% CI 1.07–3.00). Even after Bonferroni correction, the associations were observed still significantly in Asians under the two models (AA vs. GG OR1.91, 95% CI 1.31–2.80, *P* = 0.0008; AA+AG vs. GG OR 1.54, 95% CI 1.18–2.01, *P* = 0.001).

**Conclusion:**

This meta-analysis indicates that IL10 −1082 A/G polymorphism is associated with IS susceptibility in Asians and the −1082 A allele may increase risk of IS in Asians. Considering the sample size is small and between-study heterogeneity is remarkable, more studies with subtle design are warranted in future.

## Introduction

Ischemic stroke (IS) is a major cause of adult disability and death in the world[1], which is a heterogeneous multifactorial disease associated with genetic and environmental factors[2]. During the past few years, more and more evidence showed that inflammatory molecules and the genetic variation of the genes which encoded these inflammatory cytokines might take part in the pathogenesis of stroke [3]. Inflammatory mechanisms may not only play important roles in the manifestation and development of IS, but also may be vulnerable to IS in time via accumulation of atherosclerotic disease and maintain of atrial fibrillation [4]. Several candidate genes of inflammatory cytokines are implicated in the pathogenesis of IS, one of which is interleukin −10 (IL-10).

IL-10(Gene ID: 3586) is a multifunctional cytokine with anti-inflammatory properties, which has been showed involving in the inflammatory process of IS[5]. The human IL-10 gene is located on chromosome 1q31-32, in which some polymorphisms have been found in the promoter region, such as, −1082A/G (rs1800896), −592C/A (rs1800872) and −829C/T (rs1800871) [6]. The −1082A/G (also named as −1087A/G in some studies) polymorphism could affect IL-10 production [7]. And it is believed that the A/G substitution is relevant to low/high amount of IL-10 secretion, respectively [7].

Emerging studies have reported the associations between −1082G/A polymorphism in IL-10 gene and IS risk[3], [8], [9], [10], [11], [12], [13], but the results are inconclusive. Given that a single study may be too underpowered to provide reliable conclusion owing to relatively small sample size, we performed this meta-analysis to estimate the association between IL10 −1082A/G polymorphism and IS susceptibility more precisely.

## Materials and Methods

### Search Strategy

This meta-analysis conformed to the Preferred Reporting Items for Systematic Reviews and Meta-analyses (PRISMA) criteria [14]. We carried out systematic literature searches in PubMed, BIOSIS Previews, Science Direct, Chinese Biomedical Database (http://sinomed.imicams.ac.cn/index.jsp), Weipu database (http://cstj.cqvip.com/), Chinese National Knowledge Infrastructure (http://dlib3.cnki.net/kns50/),and WANFANG Database (http://g.wanfangdata.com.cn/) up to 10th October 2013 to identify relevant studies, using the following key words: (“interleukin-10” OR “interleukin 10” OR “IL-10” OR “IL10”) and (stroke OR “cerebrovascular accident” OR “cerebral ischemia” OR “cerebral infarction”) and (“polymorphism” OR “mutation” OR “genotype” OR “allele” OR “variation” OR “variant”). In addition, hand searching of the references in selected literatures and the abstracts presented at relevant conferences were performed for other potential related studies. Languages were limited to English and Chinese.

### Selection Criteria

Studies meeting the following criteria were included:(1) the study should evaluate the relationship between IL-10 gene −1082A/G polymorphism and IS risk;(2) the study had to be a case-control design; (3) genotype distributions in both cases and controls were available for calculating an odds ratio (OR) with 95% confidence interval (CI); (4)Computed tomographic(CT) or magnetic resonance imaging(MRI) were used to assess the diagnosis of IS. The following were exclusion criteria: (1) reviews, abstracts or animal studies; (2)studies were not relevant to IL-10 gene −1082A/G polymorphism or IS; (3) studies did not report genotype frequencies;(4) the design were based on sibling pairs or family. If studies were repeated or overlapped publications, the most complete one was included. If studies did not report detailed data, we would get in touch with authors to obtain the relevant information.

### Data Extraction

Two investigators (Jin and Li) reviewed and extracted data independently in accordance with the inclusion criteria. The results were compared, and if any disagreement appeared, a third investigator (Peng) was invited to evaluate such studies, then the discrepancy was resolved by discussion. The following information were extracted: the name of first author, year of publication, country (ethnicity), diagnostic criteria of IS, study design, sample size, allele numbers and genotype distributions in cases and controls.

### Quality Assessment

The quality of included studies were assessed by 2 investigators (Jin and Li) independently on the basis of Newcastle-Ottawa Scale (NOS)[15] which consisted of three aspects: selection, comparability, and exposure, and each satisfactory answer received one point. Studies with a score equal to or higher than five were regarded as of high quality.

### Statistical Analysis

Pooled ORs with corresponding 95% CIs were calculated to evaluate the strength of relationship between IL 10 gene −1082 A/G polymorphism and IS risk under the following five genetic models: the allele model (A vs. G), the homozygote model (AA vs. GG), the heterozygote model (AG vs. GG), the dominant model (AA+AG vs. GG), and the recessive model (AA vs. AG+GG). Z-test was used to assess the significance of the pooled OR, in which *P*<0.05 was considered as statistically significant. The Q-test and I^2^-statistics were employed for evaluating the between-study heterogeneity, which was considered as significant when *P_Q_*≤0.10 or I^2^>50%[16]. Then, the overall or pooled OR was obtained by a random-effect (DerSimonian-Laird method)[17] or a fixed-effect model (Mantel- Haenszel method)[18] in the presence (*P_Q_*≤0.10 or I^2^>50%) or absence (*P_Q_*>0.10 or I^2^≤50%) of heterogeneity, respectively. Furthermore,to explore the sources of heterogeneity, we conducted subgroup analysis based on ethnicity, source of controls,respectively. Moreover, Bonferroni method, controlling for false positive error rate, was utilized to adjust for multiple comparisons. As we performed multiple comparisons in this meta-analysis for 25 times, the *P* value which was less than 0.05/25 (0.002) indicated statistical significance after Bonferroni correction. To validate the reliability of the results, sensitivity analysis was performed though omitting one case–control study each time, as well as limiting this meta-analysis to studies which were conformed to HWE. HWE of genotype distribution in the controls of included studies was conducted using an online program (http://ihg2.helmholtz-muenchen.de/cgi-bin/hw/hwa1.pl), and *P*<0.05 was considered significantly deviating from HWE. Publication bias was evaluated by visual inspection of symmetry of Begg's funnel plot and assessment of Egger's test[19] (*P*<0.05 was regarded as representative of statistical significance). All statistical analyses were performed using software RevMan 5.1 and STATA 11.0.

## Results

### Study Characteristics

**Figure 1:**
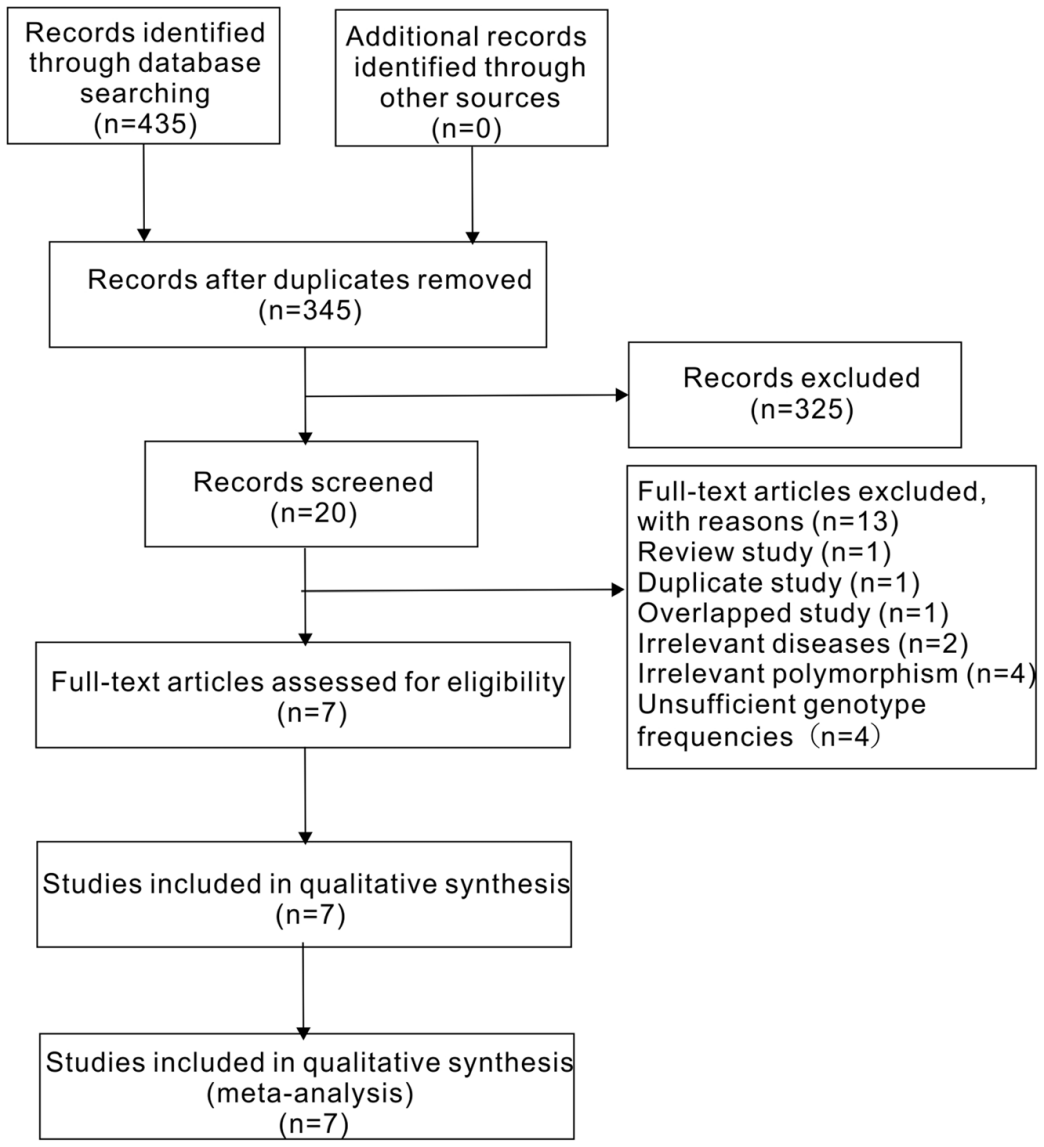
Flow diagram of the study selection process.

Figure 1 displayed the selection process of this study. A total of 435 literatures were identified after an initial search. Of these studies, the first screening excluded 415 citations based on inclusion criteria, leaving 20 articles for further selection. Among the remaining 20 literatures, 2 explored other diseases instead of IS[20], [21]; 4 researched other polymorphisms of IL-10 gene[22], [23], [24], [25]; 2 reported overlapped data [4], [8], then the one with more complete information was included[8]; 1 was a duplicate study[26];1 was a review study[27],4 did not have sufficient genotype frequencies[28], [29], [30], [31].Finally, a total of 7 studies were included in our meta-analysis [3], [8], [9], [10], [11], [12], [13],consisting of 1533 cases and 1227 controls.

The detailed characteristics of the included studies are listed in Table 1.

**Table 1:** Characteristics of studies included in the meta-analysis.

Study	Ethnicity	Study design	Control source	Genotype distribution(case/control)			HWE	NOS
				AA	AG	GG	(*P*)	
Marousi2011[8]	Greece	Case-control	Hospital based	47/53	71/71	27/21	0.72	6
Munshi2010[3]	Indian	Case-control	Population based	92/63	241/218	147/189	0.99	6
Tuttolomondo2012[9]	Caucasian	Case-control	Hospital based	58/20	14/17	24/11	0.07	6
Jin 2011[11]	Chinese	Case-control	Hospital based	161/78	27/12	1/2	0.09	7
Zhang2007[12]	Chinses	Case-control	Hospital based	202/120	2/11	0/0	0.61	6
Lin 2009[13]	Chinese	Case-control	Hospital based	153/83	28/32	0/0	0.08	6
Sultana 2011[10]	Indian	Case-control	Population based	154/163	44/47	40/16	0.000	7

All included studies were of high quality as the NOS score of each one was higher than 5 points and the genotype distributions in all controls were consistent with HWE except 1 study[10].All the 7 eligible studies were case-control studies, 2 of them were in a population-based design[3], [10], the remaining were hospital-based[8], [9], [11], [12], [13]. All studies used stroke-free people as controls except 1 study[12] which recruited healthy people as controls. All the cases were recruited from hospitalized patients and had a brain CT or MRI to assess the diagnosis of IS. 1 study only involved first-ever stroke patients[13], 2 studies included first-ever and recurrent strokes[3], [8], others did not describe the detailed information[9], [10], [11], [12]. All studies did not have age limitations for cases. Moreover, ethnic groups in these studies were as following: 2 were Caucasians[8], [9],2 were Indians[3], [10], and 3 were Chinese[11], [12], [13] (n = 3).

### Quantitative Synthesis: Overall study

**Figure 2:**
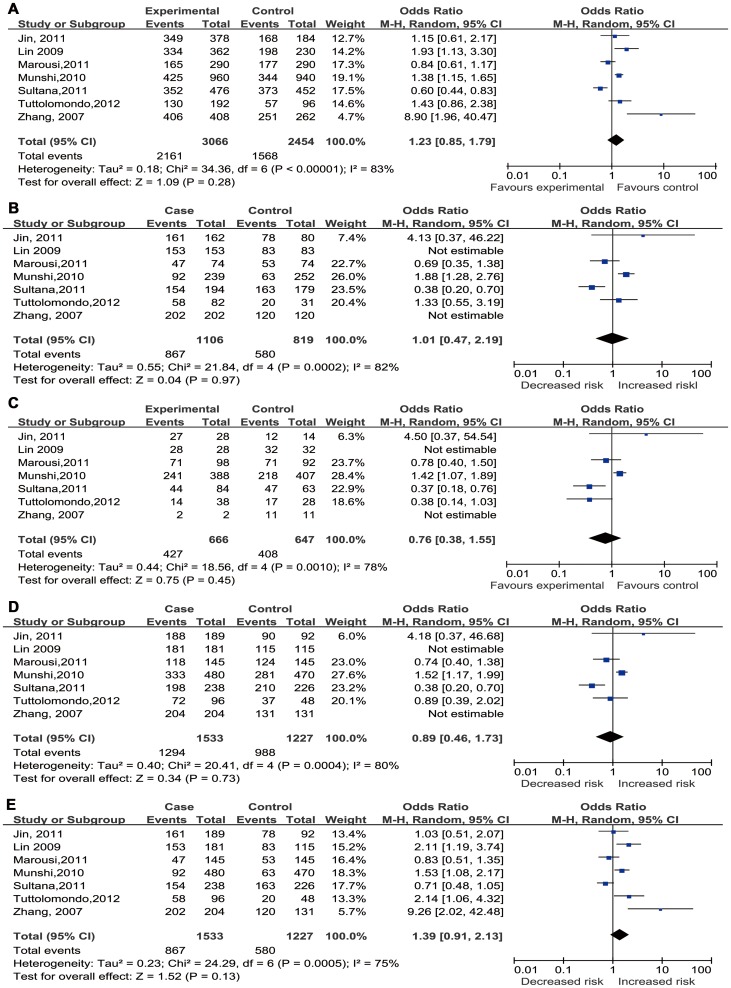
Forest plots for association between IL-10 −1082 A/G polymorphism and IS risk in different genetic models. ((A) Allele model(A vs. G); (B) Homozygote model (AA vs. GG); (C) Heterozygote model (AG vs. GG); (D) Dominant model (AA+AG vs. GG); (E) Recessive model (AA vs. AG+GG)).

As shown in Table 2 and Figure 2,significant between-study heterogeneity appeared under all genetic models for overall analysis,thus, a random-effect model was utilized to calculate the pooled estimates. Overall, no significant relationship between IL-10 −1082 A/G polymorphism and IS risk was found in all genetic models (A vs. G: OR = 1.23,95%CI = 0.85–1.79; AA vs. GG: OR = 1.01,95%CI = 0.47–2.19; AG vs. GG: OR = 0.76,95%CI = 0.38–1.55; AA+AG vs. GG: OR = 0.89,95%CI = 0.46–1.73; AA vs. AG+GG: OR = 1.39,95%CI = 0.91–2.13).

**Table 2:** Summary risk estimates for association between IL-10-1082A/G polymorphism and IS.

Comparisons	Stratifications	Studies(n)	M	Pooled estimate		Heterogeneity	
				OR(95%CI)	*P_Z_*	I^2^ (%)	*P_H_*
A vs. G	Overall	7	R	1.23(0.85, 1.79)	0.35	83	<0.0001
	Asians	5	R	1.37(0.80, 2.34)	0.26	87	<0.0001
	Non-Asians	2	R	1.06(0.63, 1.78)	0.82	66	0.09
	Hospital-based	5	R	1.47(0.90, 2.40)	0.13	73	0.006
	Population-based	2	R	0.92(0.41, 2.07)	0.84	95	<0.0001
AA vs. GG	Overall	5	R	1.01(0.47, 2.19)	0.97	82	0.0002
	Asians	3	R	1.15(0.29, 5.54)	0.84	90	<0.0001
	Non-Asians	2	F	0.88(0.51, 1.52)	0.66	25	0.25
	Hospital -based	3	F	0.96(0.57, 1.62)	0.87	29	0.25
	Population-based	2	R	0.86(0.18, 4.14)	0.94	95	<0.0001
AG vs. GG	Overall	5	R	0.76 (0.38, 1.55)	0.45	78	0.001
	Asians	3	R	0.99 (0.31, 3.19)	0.99	84	0.002
	Non-Asians	2	F	0.63 (0.36, 1.08)	0.09	28	0.24
	Hospital -based	3	F	0.69(0.41, 1.18)	0.17	46	0.16
	Population-based	2	R	0.76(0.21, 2.80)	0.69	91	0.0006
AA+AG vs.GG	Overall	5	R	0.89(0.46, 1.73)	0.73	80	0.0004
	Asians	3	R	1.02(0.30, 3.46)	0.97	89	0.0001
	Non-Asians	2	F	0.79(0.48, 1.30)	0.36	0	0.72
	Hospital -based	3	F	0.85(0.53, 1.38)	0.52	0	0.39
	Population-based	2	R	0.78(0.20, 3.06)	0.72	80	0.0004
AA vs.AG+GG	Overall	7	R	1.39(0.91, 2.13)	0.13	75	0.0005
	Asians	5	R	1.48(0.85, 2.57)	0.17	79	0.0007
	Non-Asians	2	R	1.29(0.51, 3.23)	0.59	79	0.03
	Hospital -based	5	R	1.68(0.93, 3.04)	0.09	72	0.006
	Population-based	2	R	1.05(0.49, 2.23)	0.90	88	0.004

NA, data not available;

M, Statistical model.

R, random-effects model; F, fixed-effects model.

P_Z_, P value for Z test; P_H_, P value for heterogeneity.

Notably, there was no GG genotype in either case or control group of both the Lin's study and Zhang's study[12], [13], resulting in that the two studies did not contribute to the pooled ORs in homozygote model, heterozygote model,and dominant model. In other words, in the three models, there were only 5 substantially valuable studies for overall analysis. The main results of this meta-analysis were shown in Table 2 and Figure 2.

### Subgroup analysis

To explore the sources of heterogeneity, we performed further subgroup analyses by ethnicity and source of controls respectively. Similarly, there were no significant associations in the subgroup analyses, and significant heterogeneity in most of the comparison models still existed. Table 2 showed the detailed results.

### Sensitivity Analysis

**Figure 3:**
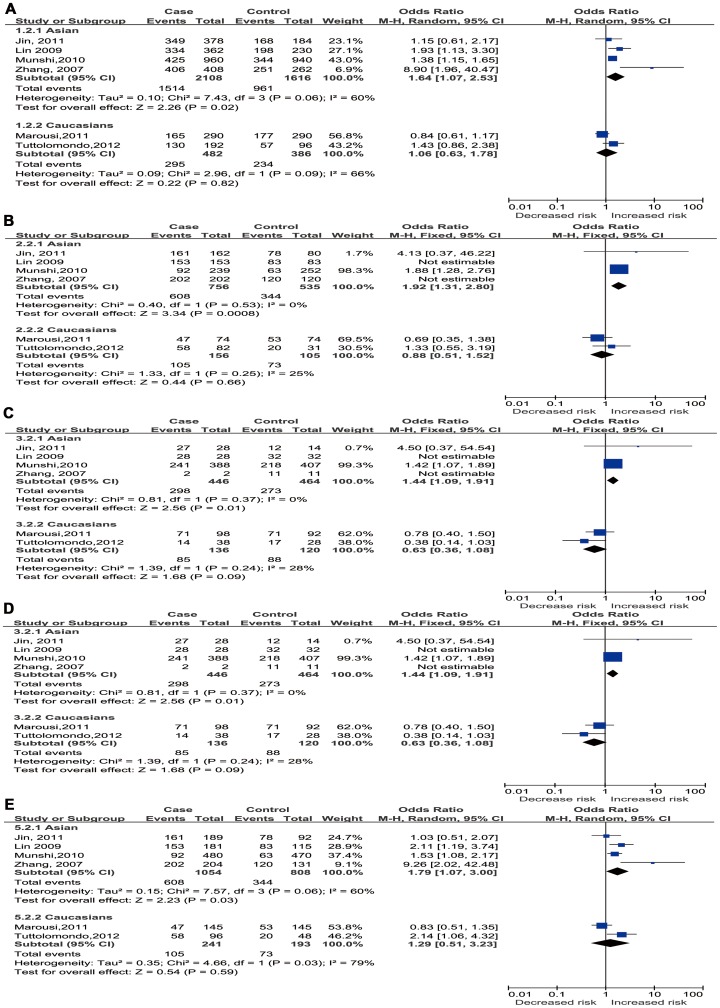
Forest plots for association between IL-10 −1082 A/G polymorphism and IS risk based on ethnicity for studies in Hardy-Weinberg equilibrium. ((A) Allele model (A vs. G); (B) Homozygote model (AA vs. GG); (C) Heterozygote model (AG vs. GG); (D) Dominant model (AA+AG vs. GG); (E) Recessive model (AA vs. AG+GG)).

Sensitivity analysis was conducted to evaluate the stability of the results. After excluding the study deviating from HWE[10], statistically significant associations were observed for Asians under all the genetic models (A VS.G: OR 1.64, 95% CI 1.07–2.53, *P* = 0.02; AA vs. GG: OR 1.91, 95% CI 1.31–2.80,*P* = 0.0008; AG vs. GG: OR1.44, 95% CI 1.09–1.91,*P* = 0.01 AA+AG vs. GG: OR1.54, 95% CI 1.18–2.01,*P* = 0.001,AA VS. AG+GG: OR 1.79, 95% CI 1.07–3.00, *P* = 0.03, whereas the corresponding pooled ORs of total available studies which meant overall analysis for all ethnicities were not materially altered (data not shown), except in recessive model(AA VS. AG+GG OR 1.58, 95% CI 1.04–2.42, *P* = 0.03). However, after Bonferronic correction for multiple testing, the associations revealed still significantly in Asian subgroup under homozygote and dominant models (AA vs. GG: OR 1.91, 95% CI 1.31–2.80, *P* = 0.0008; AA+AG vs. GG: OR1.54, *P* = 0.001). The results indicated that the homozygote AA and A allele carriers (AA+AG) had nearly a 91% and 54% increased risk of IS respectively, when compared with the homozygote GG in Asians. In particular, removing the study which was deviated from HWE eliminated the heterogeneity in subgroup analysis of Asians in homozygote, heterozygote, and dominant models, but not in the allele and recessive models and the pooling analysis (The results were shown in Table 3 and Figure 3.).Moreover, omitting the other 6 eligible studies one by one, the corresponding pooled ORs in overall comparisons and subgroup analysis were not significantly changed and the significant heterogeneity between studies still existed (data not shown).

**Table 3:** Sensitivity analysis: Study deviated from HWE were excluded in Asians under all models and for overall studies in recessive model.

Comparisons	Stratifications	Studies(n)	M	Pooled estimate		Heterogeneity	
				OR(95%CI)	*P_Z_*	I^2^ (%)	*P_H_*
A vs. G	Asians	5	R	1.37(0.80, 2.34)	0.26	87	<0.0001
	SA-A	4	R	1.64(1.07, 2.34)	0.02	60	0.02
AA vs. GG	Asians	5	R	1.15(0.29, 5.54)	0.84	90	<0.0001
	SA-A	4	F	1.91(1.31, 2.80)	0.0008*	0	0.53
AG vs. GG	Asians	5	R	0.99 (0.31, 3.19)	0.99	84	0.002
	SA-A	4	F	1.44 (1.09, 1.91)	0.01	0	0.37
AA+AG vs.GG	Asians	5	R	1.02 (0.30, 3.46)	0.97	89	0.0001
	SA-A	4	F	1.54(1.18, 2.01)	0.001*	0	0.42
AA vs.AG+GG	Overall	7	R	1.39(0.91, 2.13)	0.13	75	0.0005
	SA-O	6	R	1.58(1.04, 2.42)	0.03	66	0.01
	Asians	5	R	1.48(0.85, 2.57)	0.17	79	0.0007
	SA-A	4	R	1.79(1.07, 3.00)	0.03	60	0.03

M, Statistical model.

R, random-effects model; F, fixed-effects model.

P_Z_, P value for Z test; P_H_, P value for heterogeneity.

SA-A: Sensitivity analysis (Study deviated from HWE were exclude).in Asians.

SA-O: Sensitivity analysis (Study deviated from HWE were exclude).in overall studies.

^*^:the association is sill significant after Bonferronic correction for multiple testing.

### Publication Bias

**Figure 4:**
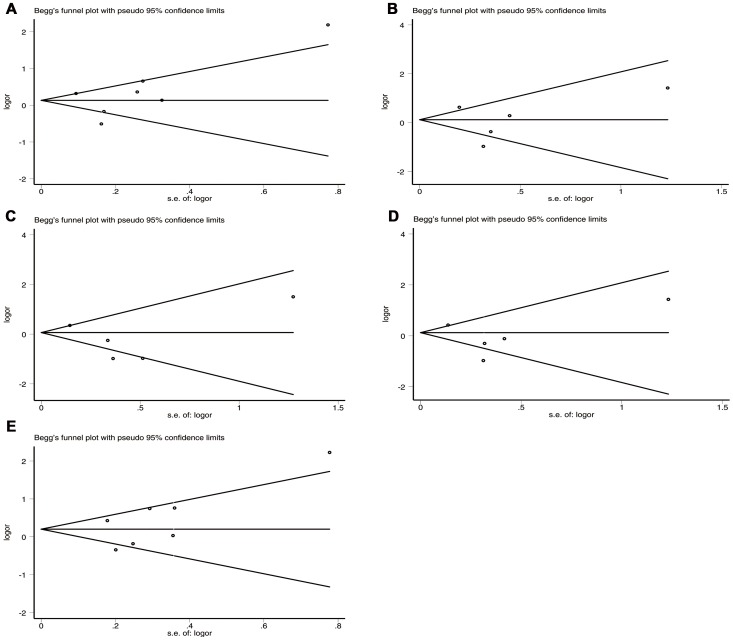
Begg's funnel plots for IL-10 −1082 A/G polymorphism and IS risk. ((A) Allele model (A vs. G); (B) Homozygote model (AA vs. GG); (C) Heterozygote model(AG vs. GG); (D) Dominant model (AA+AG vs. GG); (E) Recessive model (AA vs. AG+GG)).

No obvious visual asymmetry was observed in Begg's funnel plots, and the results of Egger's test revealed no statistical evidence for publication bias among studies (*P* = 0.632 for allele model; *P* = 0.773 for homozygote model; *P* = 0.384 for heterozygote model; *P* = 0.503 for dominant model; *P* = 0.202 for recessive model) (Table 4 and Figure 4).

**Table 4:** Publication bias tests for association between IL-10-1082A/G polymorphism and IS.

Comparisons	Egger test		Begg test
	*P* value	95%CI	*P* value
A vs. G	0.632	(−4.045, 6.047)	0.548
AA vs. GG	0.773	(−9.428, 7.727)	0.806
AG vs. GG	0.384	(−7.216, 3.719)	0.806
AA+AG vs. GG	0.503	(−7.828, 4.812)	0.806
GG vs. AG+AA	0.202	(−2.163, 7.922)	0.133

## Discussion

It is now accepted that genetics and environmental factors contribute to IS susceptibility and outcome. Similarly, the inflammation reaction is also relevant to IS. IL-10 is a potent anti-inflammatory cytokine with multiple functions taking part in inflammation reaction as well as the development of IS. Recently, the associations between IL-10 gene −1082 A/G polymorphism and the risk of IS have been intensively investigated, however, the results are inconsistent. Therefore, we designed this meta-analysis to draw a more precise conclusion for the association between IL-10 −1082 A/G polymorphism and IS risk.

In this meta-analysis, no association of the IL-10 −1082A/G polymorphism with IS risk was found under all comparisons, and in subgroup analysis by ethnicity or source of controls. However, for Asians, after excluding the study deviating from HWE[10], the data indicated that IL-10 A allele was associated with increased risk of IS in Asians. The inconsistent outcome between Asians on subgroup analysis with overall estimates partly caused by genetic diversity in different ethnicities. In addition, the different subtypes of ischemic stroke may contribute to the conflicting results,for stroke in Asians is more often due to intracranial atherosclerosis than in other populations. Furthermore, as IS is a multifactorial disease, except genetic factors, environmental factors also take important parts in IS etiology. Thus, this discrepancy may also attribute to other environmental factors, such as different geographic distribution economic status, climate, lifestyle, diet,and so on. Importantly, there is currently no consensus for whether to include studies deviating from HWE. But if the results are different between including or excluding studies deviating from HWE, it is suggested that the analysis without studies departed from HWE may be more valid [32].

Significant between-study heterogeneity displayed among all comparison models. Considering that the diversity in design, difference of ethnicity, sample sizes, and measurement errors may contribute to common sources of heterogeneity [33], we conducted the subgroup analysis by ethnicity and control of sources trying to clarify the sources of heterogeneity. Unluckily, we did not effectively eliminate the heterogeneity, indicating us that all above factors should be taken into consideration. In addition, other factors such as subtype of IS, gender distribution, past medical history, personal history and so on, might also be responsible for the heterogeneity. Notably, after removing the study deviating from HWE, the heterogeneity were removed for subgroup analysis in Asians under homozygote, heterozygote, and dominant models, suggesting that the study deviating from HWE[10] was the main source of heterogeneity in the three models for Asians.

As far as we know, this is the first comprehensive meta-analysis exploring the association between IL-10 −1082 A/G polymorphism and IS risk up to now, which involved Caucasian, Indian and Chinese populations. In addition, more studies were included in our study than a recently published meta-analysis concerning −1082A/G polymorphism and IS risk only in South Asians[27]. Our meta-analysis also has some advantages. Firstly, the search and selection studies were conducted strictly. Secondly, the results of NOS indicated that the included studies were credible. Thirdly, no evidence of publication bias was found by Begg's funnel plot and Egger's test. Fourthly, multiple testing to adjust for multiple comparisons was performed which could reduce the type I error rate. In addition, we performed sensitive analysis by excluding studies deviating from HWE, considering that deviations from HWE in healthy populations may be a sign of selection bias or population stratification[34].

Despite of the advantages mentioned above, the current study has some inevitable limitations that should be acknowledged. First, there was significant heterogeneity among included studies. Even though we used the random-effects model to calculate pool ORs, the precision of outcome would be affected. Second, owing to limiting detailed information such as lacking of subtyping of the ischemic strokes in individual study, we failed to perform further subgroup analysis to adjust these possible confounders. Third, the sample size in individual included studies was respectively small, especially in homozygote, heterozygote and dominant models. Forth, only English and Chinese language studies were included in this meta-analysis which might have led to bias. Despite of conducting an exhaustive search for eligible studies, some relevant studies so called “grey literatures” might be still missed. Fifth, similar to a case–control study, meta-analysis was a retrospective study, which might lead to recall bias. Last but not the least, this study only explored one variation in IL-10 gene, which ignored such gene–gene and gene-environmental interactions.

In conclusion,this meta-analysis indicates that IL- 10 gene −1082 A/G polymorphism is associated with IS susceptibility in Asians and the −1082 A allele may increase risk of IS in Asian populations. However, considering the limitations mentioned above, more well designed studies with adequately sized populations are needed in future.

## Supporting Information

Checklist S1 **PRISMA 2009 Checklist.**
(DOC)
